# Porous Poly(2-hydroxyethyl methacrylate) Hydrogel Scaffolds for Tissue Engineering: Influence of Crosslinking Systems and Silk Sericin Concentration on Scaffold Properties

**DOI:** 10.3390/polym15204052

**Published:** 2023-10-11

**Authors:** Nantaprapa Tuancharoensri, Sukhonthamat Sonjan, Sudarat Promkrainit, Jinjutha Daengmankhong, Preeyawass Phimnuan, Sararat Mahasaranon, Jirapas Jongjitwimol, Pensri Charoensit, Gareth M. Ross, Céline Viennet, Jarupa Viyoch, Sukunya Ross

**Affiliations:** 1 Biopolymer Group, Department of Chemistry, Faculty of Science, Naresuan University, Phitsanulok 65000, Thailandsararatm@nu.ac.th (S.M.); gareth@nu.ac.th (G.M.R.); 2 Department of Pharmaceutical Technology, Center of Excellence for Innovation in Chemistry, Faculty of Pharmaceutical Sciences, Naresuan University, Phitsanulok 65000, Thailandpensric@nu.ac.th (P.C.);; 3 Center of Excellence in Biomaterials, Faculty of Science, Naresuan University, Phitsanulok 65000, Thailand; jirapasj@nu.ac.th; 4 Biomedical Sciences Program, Department of Medical Technology, Faculty of Allied Health Sciences, Naresuan University, Phitsanulok 65000, Thailand; 5 UMR 1098 RIGHT INSERM EFS FC, DImaCell Imaging Resource Center, University of Franche-Comté, 25000 Besançon, France

**Keywords:** porous scaffolds, poly(2-hydroxyethyl methacrylate), silk sericin, crosslinking

## Abstract

Tailored porous structures of poly(2-hydroxyethyl methacrylate) (PHEMA) and silk sericin (SS) were used to create porous hydrogel scaffolds using two distinct crosslinking systems. These structures were designed to closely mimic the porous nature of the native extracellular matrix. Conventional free radical polymerization of 2-hydroxyethyl methacrylate (HEMA) was performed in the presence of different concentrations of SS (1.25, 2.50, 5.00% *w*/*v*) with two crosslinking systems. A chemical crosslinking system with *N*’*N*-methylene bisacrylamide (MBAAm) and a physical crosslinking system with dimethylurea (DMU) were used: C-PHEMA/SS (crosslinked using MBAAm) and C-PHEMA/pC-SS (crosslinked using MBAAm and DMU). The focus of this study was on investigating the impact of these crosslinking methods on various properties of the scaffolds, including pore size, pore characteristics, polymerization time, morphology, molecular interaction, in vitro degradation, thermal properties, and in vitro cytotoxicity. The various crosslinked networks were found to appreciably influence the properties of the scaffolds, especially the pore sizes, in which smaller sizes and higher numbers of pores with high regularity were seen in C-PHEMA/1.25 pC-SS (17 ± 2 μm) than in C-PHEMA/1.25 SS (34 ± 3 μm). Semi-interpenetrating networks were created by crosslinking PHEMA-MBAAm-PHEMA while incorporating free protein molecules of SS within the networks. The additional crosslinking step involving DMU occurred through hydrogen bonding of the -C=O and -N-H groups with the SS, resulting in the simultaneous incorporation of DMU and SS within the PHEMA networks. As a consequence of this process, the scaffold C-PHEMA/pC-SS exhibited smaller pore sizes compared to scaffolds without DMU crosslinking. Moreover, the incorporation of higher loadings of SS led to even smaller pore sizes. Additionally, the gelation time of C-PHEMA/pC-SS was delayed due to the presence of DMU in the crosslinking system. Both porous hydrogel scaffolds, C-PHEMA/pC-SS and PHEMA, were found to be non-cytotoxic to the normal human skin dermal fibroblast cell line (NHDF cells). This promising result indicates that these hydrogel scaffolds have potential for use in tissue engineering applications.

## 1. Introduction

Scaffolds utilized in tissue engineering have gained considerable attention among researchers as they allow for the development of new materials suitable for biomedical applications and drug delivery systems. Various scaffold properties, such as biocompatibility, stability, mechanical strength, cellular uptake, immune compatibility, and pore architecture, play a crucial role in enhancing scaffold efficiency. In this study, particular emphasis was placed on the pore architecture, and the scaffold was fabricated in a three-dimensional (3D) porous structure with interconnected pores to closely resemble the native extracellular matrix (ECM). Generally, 3D porous scaffolds are designed to provide support for the growth and function of cells, tissues, or organs, making them suitable for applications in drug delivery [1], wound healing [2], and tissue engineering, including applications in skin, dental, and bone regeneration [3,4]. Specifically, the suitable porosity of scaffolds is crucial to controlling cell addition, migration, proliferation, nutrient diffusion, and differentiation; the filtration of natural or engineered cells [5]; the diffusion of nutrients and oxygen; and waste removal [6]. Porosity can be adjusted by manipulating various variables during the scaffold fabrication process, such as types of materials, crosslinkers and solvents, polymer concentration, and types of fabrication methods (such as solvent casting, gas foaming, electrospinning, 3D-printing, emulsion-freezing, and freeze-drying).

Hydrogels have gained substantial popularity as scaffolds in tissue engineering, primarily because of their excellent biocompatibility, soft-tissue-like properties, and flexibility. They are crosslinked hydrophilic polymer networks, generated through either chemical or physical interactions, that enable the storage of large amounts of water or biological fluid in their 3D networks as well as the permeability of oxygen, nutrients, and water-soluble active agents [7]. The properties of hydrogels, such as pore size distribution, mechanical properties, and degradation behavior, can be adjusted by using different monomers, crosslinkers, and fabrication methods. The hydrogels used in biomedical applications can be made from natural polymers such as silk fibroin [8,9,10], silk sericin [11,12,13,14], collagen [15], gelatin [16], chitosan [17], and alginate [4] or synthetic polymers such as poly(2-hydroxyethyl methacrylate) (PHEMA) [18,19,20], poly(ethylene glycol) (PEG) [21], poly(vinyl alcohol) (PVA) [22], poly(N-hydroxyethyl acrylamide) (HEA) [23], poly(N-vinyl caprolactam) (PNVCL) [24], and poly(N-isopropyl acrylamide) (pNIPAAm) [25]. 

Amidst the array of polymers stated earlier, PHEMA stands out as a versatile option for designing and fabricating polymeric porous hydrogel scaffolds in this study. PHEMA has been used in various fields such as contact lenses, drug delivery systems, tissue engineering scaffolds, and biomedical applications, as it is biocompatible, water-stable, hydrophilic and an optically transparent material [26,27,28,29]. For example, interpenetrating network (IPN) hydrogels of P(HEMA-co-AM)/PVA were synthesized using one-pot microwave-assisted and conventional thermally heated methods as an efficient adsorbent with high water swelling, high porosity, and good mechanical properties [30]. The alginate-PHEMA hydrogel was produced through a hierarchical nested-network structure, which includes a crosslinked alginate hydrogel with a dense interstitial PHEMA network formed within the pores of the alginate skeleton network [18]. A porous, semi-IPN network structure and temperature-sensitive of PNIPAm-PHEMA had excellent swelling and degradation ability and a highly perforated porous structure [31]. 

Silk sericin, a natural protein composed of 18 amino acids, including up to 32% of essential amino acids such as serine, glycine, aspartic acid, glutamic acid, threonine, and tyrosine, consist of hydroxyl, carboxyl, and amino functional groups, enabling sericin to dissolve in hydrophilic solvents like water and react with other materials. It was reported as a non-toxic natural material and it was able to help increase cell density and proliferation [32,33,34]. Silk sericin was fabricated into the porous structures by incorporating with PVA, and the results showed that pore diameters of 20–30 μm were suitable to promote good efficiency for skin cell proliferation and adhesion [35]. The semi-IPN porous hydrogel scaffold of PHEA/SS was also studied, and it was found that an appropriate pore size of 10–50 μm was the most suitable for fibroblast cells to grow due to the presence of a connective porous structure along with silk sericin content [36].

**Figure 1:**
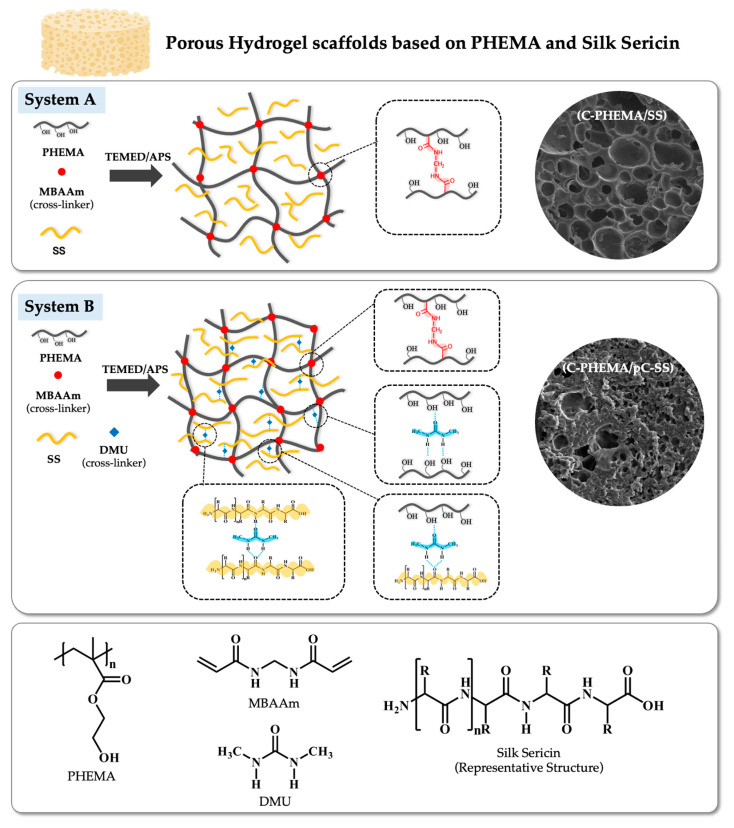
Schematic diagram of porous hydrogel scaffolds of two different fabrication systems, System A (C-PHEMA/SS, chemically crosslinked PHEMA using MBAAm in the presence of SS) and System B (C-PHEMA/pC-SS, chemically crosslinked PHEMA using MBAAm and physically crosslinked SS using DMU), showing possible molecular interactions between polymer chains and surface morphology under a scanning electron microscope, and the chemical structures of PHEMA, SS, MBAAm, and DMU.

In this research, therefore, we selected PHEMA and silk sericin (SS) as the base materials to create porous hydrogel scaffolds utilizing a lyophilization technique. For the first time, we investigated the influence of crosslinking systems and concentrations of SS on the scaffold properties. Through meticulous tailoring of the porous structures, we examined the impact of two distinct crosslinking systems, chemical crosslinking using *N*’*N*-methylene bisacrylamide (*N*’,*N*-mBAAm or MBAAm) and physical crosslinking via dimethylurea (DMU), in two different fabricated systems (refer to Figure 1). Classical free radical polymerization was used to polymerize PHEMA. The gelation time, porosity, pore size, surface morphology, degree of swelling, molecular interaction, thermal properties, in vitro degradation, and in vitro cytotoxicity of the porous scaffolds were observed. In addition, the effect of concentrations of SS (1.25, 2.50 and 5.00% *w*/*v*) on the properties of scaffolds were also investigated. This study has potential importance for designing scaffolds that mimic the native extracellular matrix (ECM) in tissue engineering, especially for skin tissue reconstruction.

## 2. Experimental Section

### 2.1. Materials 

Silk cocoons (Bombyx mori) were obtained from Tak province in the North-East region of Thailand. 2-hydroxyethyl methacrylate (HEMA), *N*’*N*-methylene bisacrylamide (MBAAm), ammonium persulfate (APS), *N*,*N*,*N*’,*N*’-tetramethyl ethylenediamine (TEMED), and *N*,*N*’-dimethylurea (DMU) were supplied by Sigma-Aldrich Co. Inc., Singapore. A normal human skin dermal fibroblast cell line (NHDF, Lot no.C-12302) was obtained from Promocell, Eppelheim, Germany. Dulbecco’s Modified Eagle Medium (DMEM), fetal bovine serum (FBS), and Trypsin/EDTA (0.25%) were purchased from Gibco (Grand Island, NY, USA). XTT (2,3,-bis(2-methoxy-4-nitro-5-sulfophenyl)-5-((phenylamino)carbonyl)-*2H*-tetrazolium hydroxide was purchased from Roche (Roche Diagnostics GmbH, Mannheim, Germany). Phosphate-buffered saline (PBS) with pH 7.4 was supplied by KEMAUS, New South Wales, Western Australia.

### 2.2. Preparation of Silk Sericin (SS) Powder via Water Degumming Process

A simple organic solvent-free process of degumming was used to prepare the silk sericin powders, following our previous research work [35,36,37,38,39]. Briefly, 20 g of silk cocoons were cut into small pieces, and then, boiled in 500 mL of DI water for 4 h. After the degumming process, the SS solution was dried into a powder in an oven at 75 °C for 13–15 h, and then, kept in a desiccator for further use. 

### 2.3. Fabrication of Porous Hydrogel Scaffold 

The porous hydrogel scaffolds were fabricated with different systems: (i) system A was crosslinked-PHEMA/SS (C-PHEMA/SS) and (ii) system B was crosslinked-PHEMA/pC-SS (C-PHEMA/pC-SS).

#### 2.3.1. System A: Crosslinked-PHEMA/SS (C-PHEMA/SS)

Silk sericin (SS) powder was fully dissolved in DI water at 100 °C in a closed system. This SS solution was cooled down to 60 °C before being placed into a round-bottom flask containing 2-hydroxyethyl methacrylate (HEMA) and continued to be mixed for 30 min before adding MBAAm (mixing for 30 min) and TEMED (mixing for 15 min) at 60 °C. This mixture solution of a total amount of 12 mL was cooled down to room temperature, and then, the solution of APS was added to start the polymerization, in which 1 mL each was added into 24-well plates. The chemical crosslinked hydrogel was fabricated, and then, purified using ethanol and DI water to remove any unreacted substances. Finally, this hydrogel was lyophilized using a freeze-dryer. Samples were left in a vacuum chamber for 24 h at −84.1 °C and 0.030 mbar to produce the porous architecture.

#### 2.3.2. System B: Crosslinked-PHEMA/pC-SS (C-PHEMA/pC-SS)

First, SS powder was completely dissolved in DI water within a closed system at 100 °C. Subsequently, dimethylurea (DMU) was introduced as a physical crosslinker (pC) to the solution. The resulting mixture of SS and pC was then combined with the solution of HEMA. After that, MBAAm and TEMED were added to the HEMA/SS/pC mixture, which was mixed for 15 min at 60 °C. Following this, the 6.50 mL mixture was allowed to cool down to room temperature before initiating the reaction by adding the solution of APS, in which 1 mL each was added into 24-well plates. Once the reaction was complete, the hydrogel was purified, and the porous structure was induced using the same process as System A. 

### 2.4. Gelation Time

For gelation time, visual observations were employed to identify the transition of the viscous liquid to a self-standing hydrogel, as over time, it turned more opaque and changed color, signaling the development of a gel network. The period of polymerization time required for gel formation after the addition of the initiator into the mixture until the formation of solid formation was recorded. Each sample was polymerized, and then, the time recorded and averaged over six repeats. Despite the inherent subjectivity and limitations of visual observation, it is an indispensable tool in hydrogel research. Yet, it is essential to acknowledge a potential 5 s margin of error in the data set.

### 2.5. Characterizations of Porous Hydrogel Scaffolds

#### 2.5.1. Morphology via SEM

The morphology of the surface and cross-sectioned surface of the porous hydrogel scaffolds were observed using a Scanning Electron Microscope (SEM, model Leo 1455VP, CARL ZEISS Co., Ltd., Oberkochen, Germany). The samples were fixed on metal stubs with carbon tape and coated via sputtering with 40–60 nm of gold before testing via SEM at 20 kV at a magnification of 200×. The experimental set up was conducted in triplicate for each sample.

#### 2.5.2. Assessment of Molecular Interaction via FT-IR

A Fourier Transform Infrared Spectrometer (FT-IR, Perkin Elmer, model Spectrum GX at 400–4000 cm^−1^) was used to study the molecular interaction of crosslinked structures of the porous hydrogel scaffolds. The samples were dried before testing in attenuated total reflection (ATR) mode.

#### 2.5.3. Assessment of Thermal Properties via DSC

Differential scanning calorimetry (DSC, Mettler model DSC1) was used to study the thermal properties of the hydrogel scaffolds: glass transition temperature (T_g_) and melting temperature (T_m_). Samples were cut and weighed to approximately 5–10 mg in an aluminum pan before testing with a heating and cooling rate of 10 °C/min under a nitrogen atmosphere in the following 3 steps: 1. heating from 25 °C to 250 °C, 2. cooling from 250 °C to −25 °C, and 3. heating from −25 °C to 25 °C. 

#### 2.5.4. Swelling Ratio 

The swelling ratios of the porous hydrogel scaffolds were measured according to a conventional gravimetric method. The samples were immersed in DI water at 37 °C. The swelling ratio was calculated by monitoring the ratio of the wet weight and dry weight of the porous hydrogels at set time intervals, using the following equation.

$$\%\;Swelling\;ratio=\frac{W_{t}-W_{0}}{W_{0}}\times 100$$

where *W*_0_ is the weight of the dried porous hydrogel scaffold and $W_{t}$ is the weight of the fully swollen porous hydrogel scaffold. 

#### 2.5.5. In Vitro Degradation

The in vitro degradation of the porous hydrogel scaffolds was observed by soaking lyophilized porous hydrogel scaffolds (0.5 cm × 0.5 cm) in phosphate-buffered saline solution (PBS pH 7.4) in an incubator at 37 °C. The weight loss of samples (in dry state) was measured using an analytical balance to 3 decimal places (0.001) at 7, 14, 21, and 28 days, with each sample tested in triplicate. 

#### 2.5.6. In Vitro Cell Cytotoxicity

The cytotoxicity assessment of the NHDF cell line for the porous hydrogel scaffolds (C-PHEMA, C-PHEMA/1.25SS, and C-PHEMA/1.25pC-SS) was carried out utilizing an XTT assay. In this methodology, the porous hydrogel scaffold samples (measuring 1.55 cm in diameter and 5 mm in thickness) were first prepared as hydrogel extracts. This was achieved by immersing the porous hydrogel scaffolds in 1 mL of serum-free DMEM and allowing them to incubate at 37 °C within a CO_2_ incubator for 24 h. The hydrogel extracts were then sterilized by passing them through a 0.2 µm filter. For the assessment of cell viability, NHDF cells (at a density of 1 × 10^4^ cells/well and with a passage number not exceeding 10) were seeded into 96-well plates. These cells were cultured in DMEM containing 10% FBS and were incubated at 37 °C in a humidified atmosphere with 5% CO_2_ for 24 h. Following this initial incubation, the culture medium was replaced with the prepared hydrogel extract, and the cells were further incubated for 24 h. Subsequent to this incubation period, the medium containing the hydrogel extract was replaced with fresh serum-free medium containing the XTT reagent, and the cells were incubated for an additional 4 h. After incubation with the XTT reagent, the absorbance of the samples was measured at 517 nm using a microplate reader. This experiment was conducted in triplicate (n = 9) for each experimental condition. The percentage of viability for the control condition (cells treated with serum-free DMEM) was set at 100%.

## 3. Results and Discussion

Two different novel porous hydrogel scaffolds were fabricated from PHEMA and SS using different fabricated systems (A and B) and concentrations of SS (1.25, 2.50, and 5.00% *w*/*v*) (Table 1). The polymerization of PHEMA occurred via free radical polymerization using TEMED (as a catalyst) and APS (as an initiator), and the formation of crosslinked-networks was achieved using *N*’,*N*-MBAAm (abbreviated as MBAAm) in both systems. Additionally, DMU was introduced as a secondary crosslinker, engaging primarily in hydrogen bonding interactions in system B. Polymerization time (reported as gelation time) and the characteristics of these two different porous hydrogel scaffolds were compared together with the pure PHEMA and crosslinked PHEMA (C-PHEMA) as control samples.

### 3.1. Gelation Time and Physical Appearance

**Figure 2:**
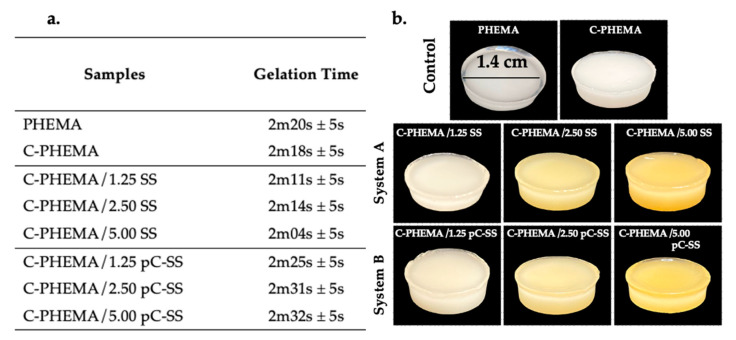
Gelation times (**a**) and physical appearance (**b**) of hydrogels before lyophilization with System A (C-PHEMA/SS) and System B (C-PHEMA/pC-SS).

Gelation time was recorded once the polymer solution had transformed into a solid structure, the initial time was after the addition of APS (initiator) into the mixed solution of either HEMA or HEMA and SS. Figure 2a,b show the gelation times and physical appearances of all hydrogel scaffolds before lyophilization. The gelation times of PHEMA and crosslinked PHEMA (C-PHEMA) are approximately 2.20 and 2.18 min, respectively. All samples made using the same mold and that have a consistent 1.4 cm diameter display different physical appearances: the PHEMA hydrogel is clear-white, while the C-PHEMA hydrogel is opaque-white.

In system A, characterized by crosslinked-PHEMA/SS, the gelation time was found to be marginally lower compared to the controlled samples. Higher concentrations of SS did not exert a substantial impact on the gelation time, but the influence of greater SS produced a stronger intensity of yellow color in the scaffolds. However, when physically adding a crosslinker (DMU) into system B, the gelation times are slightly higher compared to the control samples and system A. It is possible that this slight delay is caused by the incorporation of the additional physical crosslinking systems’ hydrogel scaffolds in system B. As depicted in Figure 1, the construction of polymer networks in system A involved the utilization of a chemical crosslinker (BMAAm) to form PHEMA. Simultaneously, the protein molecules of SS were incorporated within the polymer structure, resulting in a semi-interpenetrating network (semi-IPN) configuration. In contrast, the supplementary crosslinker (DMU) functioned through physical molecular interactions involving hydrogen bonding between the -C=O and -N-H groups, concurrently with the formation of polymer networks of PHEMA. Additionally, hydrogen bonding between PHEMA-DMU-PHEMA and PHEMA-DMU-SS (by -OH, -C=O, and -N-H groups) represented possible interactions. The physical crosslinking by DMU (pC-SS) acted as a supplementary network and slightly increased the gelation time of scaffolds in system B. Furthermore, the concentration of SS in system B exhibited similar behavior to system A, as it did not exert a meaningful effect on the gelation time. The physical appearance of all hydrogels was opaque-yellow, and the intensity of the yellow color intensified proportionally with higher loading levels of silk sericin.

### 3.2. Morphology of Porous Hydrogel Scaffolds

**Figure 3:**
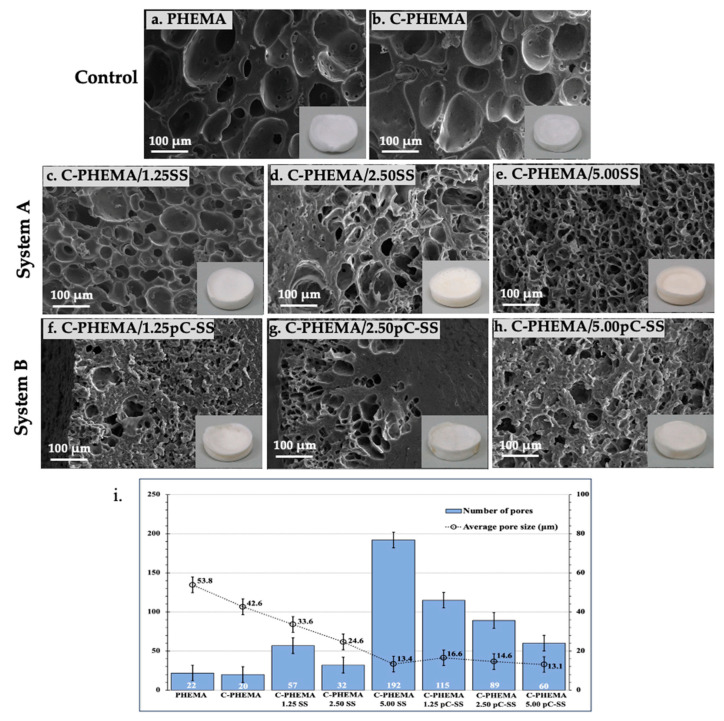
SEM images together with physical appearances of the porous hydrogel scaffolds of (**a**) PHEMA, (**b**) C-PHEMA, (**c**–**e**) System A (C-PHEMA/SS), and (**f**–**h**) System B (C-PHEMA/pC-SS) at different loadings of SS of 1.25, 2.50, and 5.00% *w*/*v*, respectively, and (**i**) bar chart of average pore size of all scaffolds, measured using ImageJ software (FIJI 1.46 version I.J 1.46r by SciMark 2.0).

The hydrogels synthesized from both system A (C-PHEMA/SS) and system B (C-PHEMA/pC-SS) were subjected to lyophilization to facilitate the formation of porous structures. The physical appearance of each scaffold, following the lyophilization process, is visually depicted in the inset pictures presented in Figure 3. Additionally, the cross-sectioned surface morphology of the scaffolds was observed using SEM. The results show that PHEMA (Figure 3a) and C-PHEMA (Figure 3b) had pore diameter size of 54 ± 3 μm and 42 ± 3 μm. The porous structure and pore diameters of all hydrogel scaffolds were dependent on the fabrication systems employed and the loading of SS. At an equivalent concentration of SS, the scaffolds produced using system A (C-PHEMA/SS) demonstrated larger pore sizes compared to those fabricated using system B (C-PHEMA/pC-SS). For example, at 1.25% *w*/*v* SS, the system A scaffold showed an average diameter of 34 ± 3 μm (Figure 3c), while the system B scaffold showed an average diameter of 17 ± 2 μm (Figure 3f). The scaffolds created using system B had fewer pores compared to those made using system A. This difference can be attributed to the rigid gel network structure in system B, which opposes the formation and expansion of water crystal [40]. In addition, the different fabrication systems resulted in different crosslinked networks, which affected the morphology and properties of the hydrogel scaffolds. 

The influence of SS loading on the pore diameter size was also investigated. It was observed that higher SS loadings led to a reduction in the diameter of the pores. For instance, in the scaffold fabricated using system A, the pore diameter sizes were measured at 34 μm, 25 μm, and 13 μm when SS was used at concentrations of 1.25% *w*/*v*, 2.50% *w/v*, and 5.00% *w*/*v*, respectively. This phenomenon can be attributed to the increased number of molecular chains of SS at higher loading levels, which enhances the interpenetration of SS within the networks. Consequently, a higher concentration of SS forming the solid network between the pores would reduce the size of the pores.

### 3.3. Swelling Ratios 

Swelling behavior is an important property for biomaterials because it is the first step in the degradation process of materials. Importantly, it helps to control the migration and growth of cells through the crosslinked network as well as the diffusion of any bioactive molecules added into the hydrogel scaffold. Crosslinking creates a network of polymer chains that can absorb more water, leading to a higher swelling ratio. The mean swelling after 24 h of porous hydrogel scaffolds based on PHEMA and SS in different fabricated systems and at different concentrations of SS is reported in DI water at 37 °C in Table 2. PHEMA without a crosslinker had the lowest swelling (approximately 100% after 24 h) compared to other samples, while the addition of MBAAm to PHEMA (C-PHEMA) promoted higher swelling (approximately 150% after 24 h). 

These results show that the swelling of all hydrogel scaffolds depended on the fabricated system and the loading of SS. The swelling amounts observed for the porous hydrogel scaffolds fabricated via system A exhibited higher values in comparison to those from system B. This discrepancy implies that the presence of dual crosslinking networks in system B, involving both chemical crosslinking through MBAAm and physical crosslinking via DMU, has an impact on the water absorption capability within their networks, leading to a lower degree of swelling for the scaffolds. In addition, the different loading of SS seems to affect the swelling properties of scaffolds, depending on the crosslinking systems. In system A, SS loading at 5% *w*/*v* promoted the highest swelling, which might be due to highest hydrophilic interaction between amino acid groups in SS and water. In system B, the utilization of 2.50% *w*/*v* of SS loading resulted in the highest swelling, demonstrating that this particular amount was well suited in relation to the DMU content. Moreover, the degree of swelling of a hydrogel scaffold also depends on its pore size and pore distribution. Larger pore sizes (as seen in Figure 3; system A) and more uniform pore distributions can lead to higher degrees of swelling. This is because larger pores allow for more water uptake, while a more uniform distribution of pores allows for water to be distributed more evenly throughout the scaffold, ultimately leading to a more pronounced swelling response.

### 3.4. In Vitro Degradation

**Figure 4:**
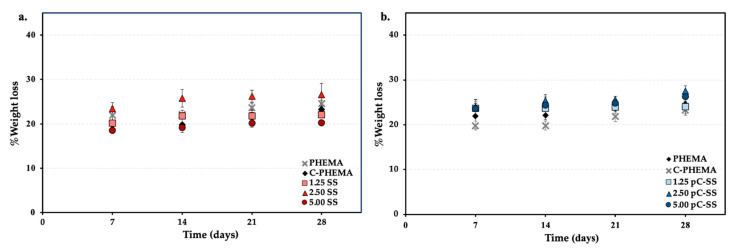
In vitro degradation of porous hydrogel scaffolds with different fabricated systems of (**a**) System A (C-PHEMA/SS) and (**b**) System B (C-PHEMA/pC-SS).

The degradation behavior of the porous hydrogel scaffolds is influenced by both the distinct fabrication systems and the quantity of silk loading, as illustrated in Figure 4. In vitro degradation was assessed by measuring the percentages of weight loss of the scaffolds, which were immersed in phosphate-buffered saline (PBS) pH 7.4 at 37 °C for 7, 14, 21, and 28 days. The result shows that the percentage of weight loss of all porous hydrogel scaffolds are similar, at approximately 20–30%. These results demonstrate that the presence of silk sericin does seem to increase the degradation of the hydrogel scaffolds by a few percent, as evidenced by the higher percentage of weight loss observed in the scaffolds containing silk sericin compared to the control samples (PHEMA and C-PHEMA). However, there was not a considerable difference observed in weight loss between the different silk loading values (1.25–5.00% *w*/*v*) tested in this study. 

### 3.5. Functional Groups

**Figure 5:**
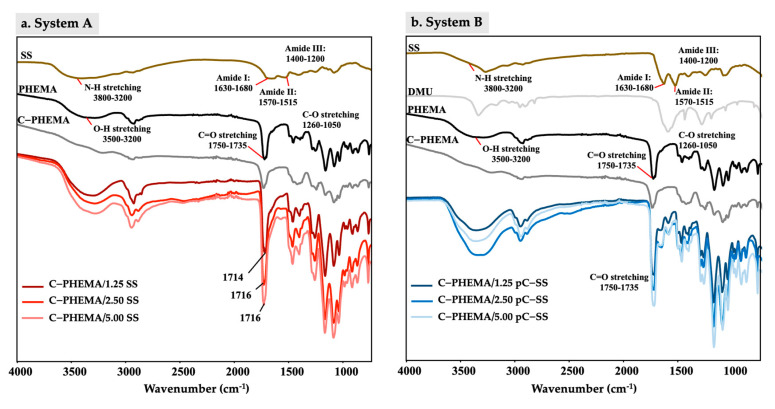
FTIR spectra of porous hydrogel scaffolds with different fabricated systems of (**a**) System A; C−PHEMA/SS and (**b**) System B; C−PHEMA/pCSS.

The porous hydrogel scaffolds were analyzed for their functional groups via FT-IR, and the results are seen in Figure 5. PHEMA without a crosslinker shows mainly the following groups: hydroxyl (-OH stretching) at 3600–3200 cm^−1^, methyl and methylene (C-H stretching) at 2847–2948 cm^−1^, ester carboxyl (C=O stretching) at 1750–1735 cm^−1^, and -C-H bending at 1451 cm^−1^. The -C=C stretching of HEMA at 1650 cm^−1^ was not observed. This can therefore confirm the successful polymerizetion of PHEMA [41]. The peak of -OH stretching shifted to a lower frequency when PHEMA was crosslinked. Silk sericin (SS) consists of 16–18 amino acid groups and has three main functional groups: Amide I (C=O stretching) at 1630–1680 cm^−1^, Amide II (N-H bending) at 1570–1515 cm^−1^, and Amide III (in phase combination of N-H in plane-bending and C-N stretching vibrations) at 1400–1200 cm^−1^ [35]. 

In system A, the characteristic peaks of C-PHEMA and SS were observed, which confirm the crosslinked structure of hydrogels containing SS molecules. However, the -OH stretching and hydrogen bonds at 3600–3200 cm^−1^ were shifted to a lower wavenumber when higher SS was added into the hydrogel scaffold. The increased number of SS chains promotes the slightly higher formation of hydrogen bonding. Consequently, this leads to a change in the electron cloud and alters the resonant frequency of the hydrogen bonds, causing them to have lower energy levels, resulting in lower frequency bands during molecular vibrations [35]. This can confirm that the hydrogel scaffold fabricated using system A promotes a semi-IPN structure with the chemical crosslinker (MBAAm) with PHEMA, and the free chains of SS (with some hydrogen bonding) are embedded in the crosslinked polymer network of PHEMA (see Figure 1, system A). 

In system B, the characteristic peaks of C-PHEMA were observed. The shifted peaks of Amide I, II, and III of SS were monitored and compared to the characteristic peaks of neat SS. In addition, broader peaks of -OH stretching and hydrogen bonds were seen when DMU was added into the system. This is because the stronger hydrogen bonding interactions cause a lowering of the electron density in the carbonyl and hydroxyl groups, and then, shifted absorption bands appear. This suggests that two possible crosslinking networks are present, one by MBAAm (a crosslinker for PHEMA) and one by DMU (a physical crosslinker for SS) (see Figure 1, system B). The physical crosslinking occurs due to the interaction of two H-atoms of the DMU molecule with the carbonyl oxygen of either another DMU or an SS molecule to promote physically crosslinked DMU or SS-DMU-SS, respectively. In addition, the hydrogen bonding between PHEMA and either DMU or SS is a possible alternative molecular interaction due to the presence of -OH and -NH in these molecules. 

### 3.6. Thermal Properties

**Figure 6:**
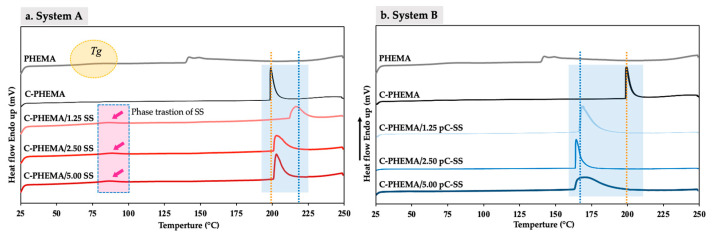
DSC thermogram of porous hydrogel scaffolds with different fabricated system of (**a**) System A (C-PHEMA/SS) and (**b**) System B (C-PHEMA/pC-SS).

The thermal properties of the hydrogel scaffolds were further investigated, and the findings are presented in Figure 6. The glass transition temperature (T_g_) of PHEMA is observed at 79 °C, whereas no distinct peaks are observed when PHEMA was crosslinked. From Figure 6a, a small phase transition of SS in the scaffolds is observed at approximately 75–85 °C, while it is not seen in other scaffolds (Figure 6b). This is attributed to the conformational change in silk protein from *β*-sheets to random coils in system A, which has free movement of SS chains as there is no crosslinking. The melting peaks of C-PHEMA/SS scaffolds (except C-PHEMA/1.25) in system A show at the same position of C-PHEMA but are broader. From Figure 6b, the melting peaks of C-PHEMA/pC-SS appear at a lower temperature and are broader than those of C-PHEMA. This is because the system takes less energy to disrupt the crystal lattice when pC-SS is present. The introduction of pC-SS disrupts the regular arrangement of C-PHEMA, resulting in a reduction in the energy required to break the bonds, and subsequently causing a decrease in the melting point. Moreover, an increase in pC-SS loading leads to a more pronounced shift and broader peaks, attributed to greater disruption of the C-PHEMA chains.

### 3.7. In Vitro Cell Cytotoxicity

**Figure 7:**
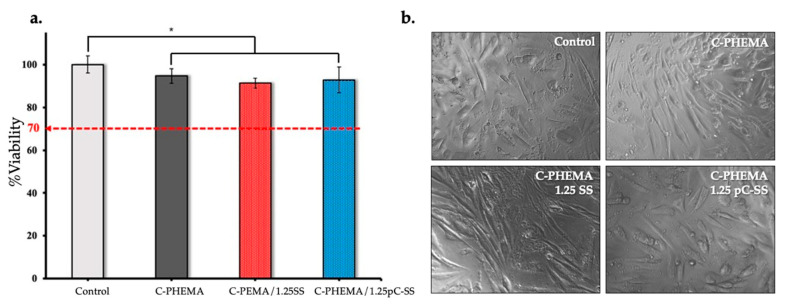
In vitro cell cytotoxicity assessment of NHDF cells for the porous hydrogel scaffolds (C-PHEMA, C-PHEMA, C-PHEMA/1.25SS, and C-PHEMA/1.25pC-SS) via XTT assay: (**a**) percentage of cell viability of NHDF cells, n = 9, * denotes *p* value < 0.05 and (**b**) optical microscopy images of NHDF cells after 24 h incubation with the extract solution of the scaffolds.

Fibroblasts are the main cells of connective tissue and they interact closely with the extracellular matrix in the repair process. Cytotoxicity is considered a pilot test and an important indicator for the toxicity evaluation of biomaterials. According to the ISO standard cytotoxicity protocol, a material inducing cell viability above 70% is considered non-cytotoxic [42]. From Figure 7, the results indicate that all of the porous hydrogel scaffold samples, including C-PHEMA, C-PHEMA/1.25SS, and C-PHEMA/1.25pC-SS, as well as the control, exhibit high cell viability (for 24 h) of over 90%, with no cytotoxic effects observed. The optical microscopic images of NHDF show normal cell morphology with an adherent spindle shape of the fibroblast phenotype, and therefore confirm the viability of cells in these hydrogel scaffolds. Based on these results, it can be inferred that the porous hydrogel scaffolds are non-cytotoxic to the NHDF cells. Therefore, these hydrogel scaffolds hold promise for potential applications in skin tissue regeneration.

## 4. Conclusions

This work aimed to explore the influence of two crosslinking systems, involving chemical crosslinking via BMAAm and physical crosslinking through DMU, on the morphology and physical properties of porous hydrogel scaffolds composed of PHEMA and SS. The pairing of the PHEMA-BMAAm-PHEMA and SS-DMU-SS crosslinking networks led to an extended gelation period and smaller, more uniform pore sizes in PHEMA/pC-SS scaffolds. Higher loadings of SS resulted in smaller-diameter pore sizes of the scaffolds. The presence of dual crosslinking networks affected the water absorption within their networks, leading to a lower degree of swelling in the scaffolds. When subjected to in vitro degradation in PBS at 37 °C, all of the porous hydrogel scaffolds exhibited a comparable percentage of weight loss, ranging roughly between 20% and 30%. However, the presence of silk sericin was observed to slightly enhance the degradation of the hydrogel scaffolds. In addition, all scaffolds were non-toxic to fibroblast cells. These results collectively suggest that crosslinking systems and concentrations of silk sericin play crucial roles in tailoring the properties of porous hydrogel scaffolds composed of PHEMA and SS. These findings provide valuable insights for designing hydrogel scaffolds with tailored properties for potential applications in tissue engineering, particularly in the context of skin tissue regeneration.

## Figures and Tables

**Table 1:** Compositions of porous hydrogel scaffolds with different fabricated systems and concentrations of SS.

System	Samples	HEMA (g)	MBAAm (%wt)	TEMED (%wt)	APS (%wt)	Silk Sericin		Total Volume (mL)
						SS (% *w*/*v*)	DMU (%wt)	
**Control**	PHEMA	2.154	-	0.50	0.25	-	-	6.50
	C-PHEMA	2.154	0.50	0.50	0.25	-	-	6.50
**A**	C-PHEMA/1.25 SS	2.154	0.50	0.50	0.25	1.25	-	6.50
	C-PHEMA/2.50 SS	2.154	0.50	0.50	0.25	2.50	-	6.50
	C-PHEMA/5.00 SS	2.154	0.50	0.50	0.25	5.00	-	6.50
**B**	C-PHEMA/1.25 pC-SS	2.154	0.50	0.50	0.25	1.25	5.57	6.50
	C-PHEMA/2.50 pC-SS	2.154	0.50	0.50	0.25	2.50	5.57	6.50
	C-PHEMA/5.00 pC-SS	2.154	0.50	0.50	0.25	5.00	5.57	6.50

**Table 2:** Mean Swelling after 24 h of porous hydrogel scaffolds with different fabricated systems.

Samples	Mean Swelling after 24 h ± SD
PHEMA	111.5 ± 0.9
C-PHEMA	159.2 ± 2.5
C-PHEMA/1.25 SS	256.8 ± 2.9
C-PHEMA/2.50 SS	232.7 ± 0.8
C-PHEMA/5.00 SS	290.6 ± 4.7
C-PHEMA/1.25 pC-SS	158.3 ± 3.8
C-PHEMA/2.50 pC-SS	240.8 ± 5.2
C-PHEMA/5.00 pC-SS	209.8 ± 3.1

## Data Availability

The raw/processed data required to reproduce these findings cannot be shared at this time as the data also form part of an ongoing study.
